# The role of breastfeeding in breast cancer prevention: a literature review

**DOI:** 10.3389/fonc.2023.1257804

**Published:** 2023-09-07

**Authors:** Yulong Chen, Pengli Jiang, Yongqin Geng

**Affiliations:** ^1^ Department of Thyroid and Breast Surgery, The Fourth People’s Hospital of Jinan, Jinan, China; ^2^ Department of Breast Surgery, The Second Hospital of Jilin University, Changchun, China; ^3^ Department of Thyroid and Breast Surgery, Central Hospital Affiliated to Shandong First Medical University, Jinan, China

**Keywords:** breastfeeding, breast cancer, mechanism, methylation, breast involution

## Abstract

Breast cancer stands as the most prevalent malignancy globally. Despite the array of treatment options, its mortality rate continues to rise annually. Thus, reevaluation of factors directly linked to breast cancer emergence is imperative, alongside the development of more effective preventive measures. Estrogen levels, profoundly tied to reproduction, play a pivotal role in breast cancer development. Speculation centers on the potential of breastfeeding to mitigate cancer risk in women. However, the precise mechanism remains elusive. Breastfeeding is a modifiable reproductive factor extensively studied. Studies highlight a direct connection between lack of breastfeeding and breast cancer emergence, potentially linked to DNA methyltransferase expression alteration, aberrant methylation levels, pregnancy-associated plasma protein-A, cellular microenvironment, and oncogenes. This study reviews recent mechanisms underlying breastfeeding’s role in reducing breast cancer incidence.

## 1 Background

Statistics from GLOBLCAN 2020 underscore breast cancer’s ascension as the world’s most prevalent cancer type, surpassing lung cancer (1). Developed regions like Western and Northern Europe exhibit nearly 88% higher breast cancer incidence rates than underdeveloped counterparts (East and Central Africa). This global shift necessitates consideration of known risk factors against the backdrop of cancer incidence rate alterations. Notably, age emerges as a primary risk factor, with older females witnessing the highest age-specific incidence rates. Average youthfulness in underdeveloped nations is shaped by aging populations and a ten-year drop in life expectancy. Economic growth in these areas corresponds with elevated life expectancies, indicating imminent cancer incidence rate increases (1, 2). Reproductive and non-reproductive factors, both influenced by economic development, comprise vital risk factors. Reduced menarche age, delayed menopause age, fewer children, and decreased breastfeeding escalate breast cancer risk (3). Improved human development often accelerates menarche onset by enhancing nutritional status, a determinant of menarche initiation age (4). Non-reproductive risk factors include obesity, with breast cancer risk doubling in overweight postmenopausal women. It’s projected that increased alcohol consumption will contribute to about 4% of diagnosed cancer cases in 2020 (5). While potential genetic or hereditary causes like BRCA1 or BRCA2 mutations account for 5-10% of breast cancers, eight out of nine cases lack affected female reproductive systems (6). Moreover, variations in breastfeeding prevalence might underlie observed differences, along with differences in the healthcare systems levels (7). While West African countries report 70% exclusive breastfeeding rates for five consecutive months, European countries average 30% (8). Despite this, even in the US, access to pumping facilities remains limited, constraining breastfeeding duration. Breastfeeding moderately affects breast cancer occurrence; Almasi-Hashiani et al. determined that 27.3% of breast cancer patients developed cancer due to inadequate breastfeeding (9). Notably, US black women exhibit lower breastfeeding rates and nearly double the triple-negative breast cancer (an aggressive subtype) incidence rates compared to white women (10). A Malaysian case-control study involving 7,663 women found that ever-breastfeeding and longer breastfeeding durations correlated with reduced breast cancer risk (11). The study further suggested that breastfeeding for under three months raised breast cancer risk, while exceeding 12 months lowered risk (12, 13). This raises speculation regarding breastfeeding’s potential to mitigate cancer risk *via* (1) breast cell differentiation, modifying them for post-production milk production, thereby reducing breast tissue vulnerability to carcinogenesis (estrogen), and (2) impeding ovulation by diminishing estrogen’s mitosis-promoting effect (14). Carcinogen secretion in breast milk and breast tissue shedding contribute to damaged DNA cell elimination, curtailing mutation responsiveness (15). However, the precise mechanism remains uncertain. Understanding how breastfeeding mechanisms impact breast cancer can identify pharmacologic preventive measures for non-breastfeeding women, curbing breast cancer prevalence and mortality. Additionally, these findings might inform novel treatment approaches. Therefore, this review consolidates recent discoveries concerning pertinent mechanisms and processes.

## 2 Breastfeeding and DNA methylation

DNA methylation, an epigenetic modification prevalent in mammals, is catalyzed by DNA methyltransferase enzymes, including DNMT1, DNMT3a, and DNMT3b (16). This alteration affects DNA’s transcriptional participation, leading to gene silencing and diminished transcriptional capacity. Elevated DNA methyltransferase content was observed in breast cancer patients (17).

Forkhead box protein A1 (Fox A1), a transcription factor, fosters luminal progenitor cell differentiation into mature luminal cells during breast development while repressing the basal phenotype (18). Highly methylated in *BRCA-1*related breast cancers (19), Fox A1’s methylation possibly stems from *BRCA-1* gene control over Fox A1 expression *via* methyltransferase inhibition (20). Conversely, BRCA-1 gene mutation or silencing abolishes this inhibitory effect, enabling Fox A1 hypermethylation, fostering breast cancer (21).

**Figure 1:**
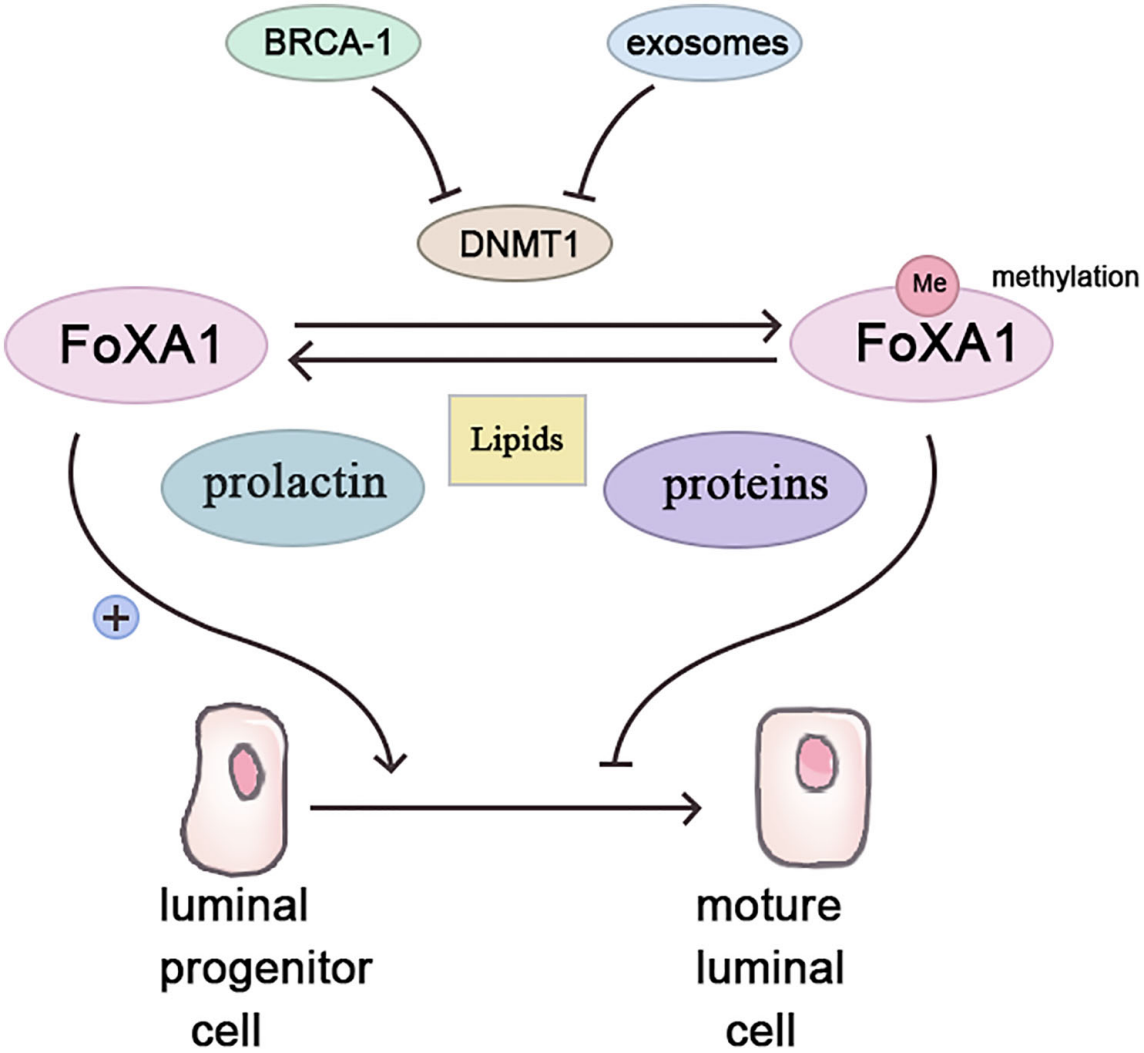
Breastfeeding inhibits the occurrence of breast cancer by affecting the methylation of FoxA1.

Contrastingly, parous women with breastfeeding history exhibit lower Fox A1 methylation, while non-breastfeeding induces Fox A1 hypermethylation akin to BRCA-1 mutation effects (21, 22). Two protective breastfeeding mechanisms include pregnancy and exosomes. Pregnancy triggers mammary epithelial cell DNA methylation for subsequent lactation readiness (23). Prolactin, during lactation, stimulates mammary epithelial cell milk protein and lipid synthesis, demethylating breastfeeding-related genes (24). Breast milk’s exosomes, small vesicles rich in various molecules, including proteins, lipids, and microRNAs, exert significant physiological and pathological influence (25). In breast tissue, exosomes govern lactation and mammary gland involution (26). Breast milk’s exosomes contain abundant mir-29s and mir-148a, downregulating methyltransferases DNMT3/b and DNMT1 (27, 28). *In vitro* studies confirm epithelial cell exosome uptake and maintenance of functionality (29). Breastfeeding’s absence potentially reduces mammary epithelial cell exosome exposure, increasing DNA methyltransferase expression and aberrant methylation (**Figure 1**).

## 3 Breastfeeding and pregnancy-associated plasma protein-A

Pregnancy-associated plasma protein-A (PAPP-A), a metzincin (30)metalloproteinase, is overexpressed in most breast cancer patients (31). Insulin-like growth factor 1 (IGF-1) safeguards mammary epithelium against apoptosis (32). Insulin-like growth factor binding protein-5 (IGFBP-5), a vital mammary gland involution regulator, impedes IGF receptor activation by sequestering IGF-1 (33). Conversely, PAPP-A hampers IGFBP-5 *via* hydrolysis, delaying breast involution and heightening pregnancy-associated breast cancer (PABC) risk (34). Collagen deposition elevates PAPP-A, enhancing IGFBP-5 cleavage, reinforcing IGF signaling, intensifying collagen deposition, thereby augmenting PABC risk (30, 34).

**Figure 2:**
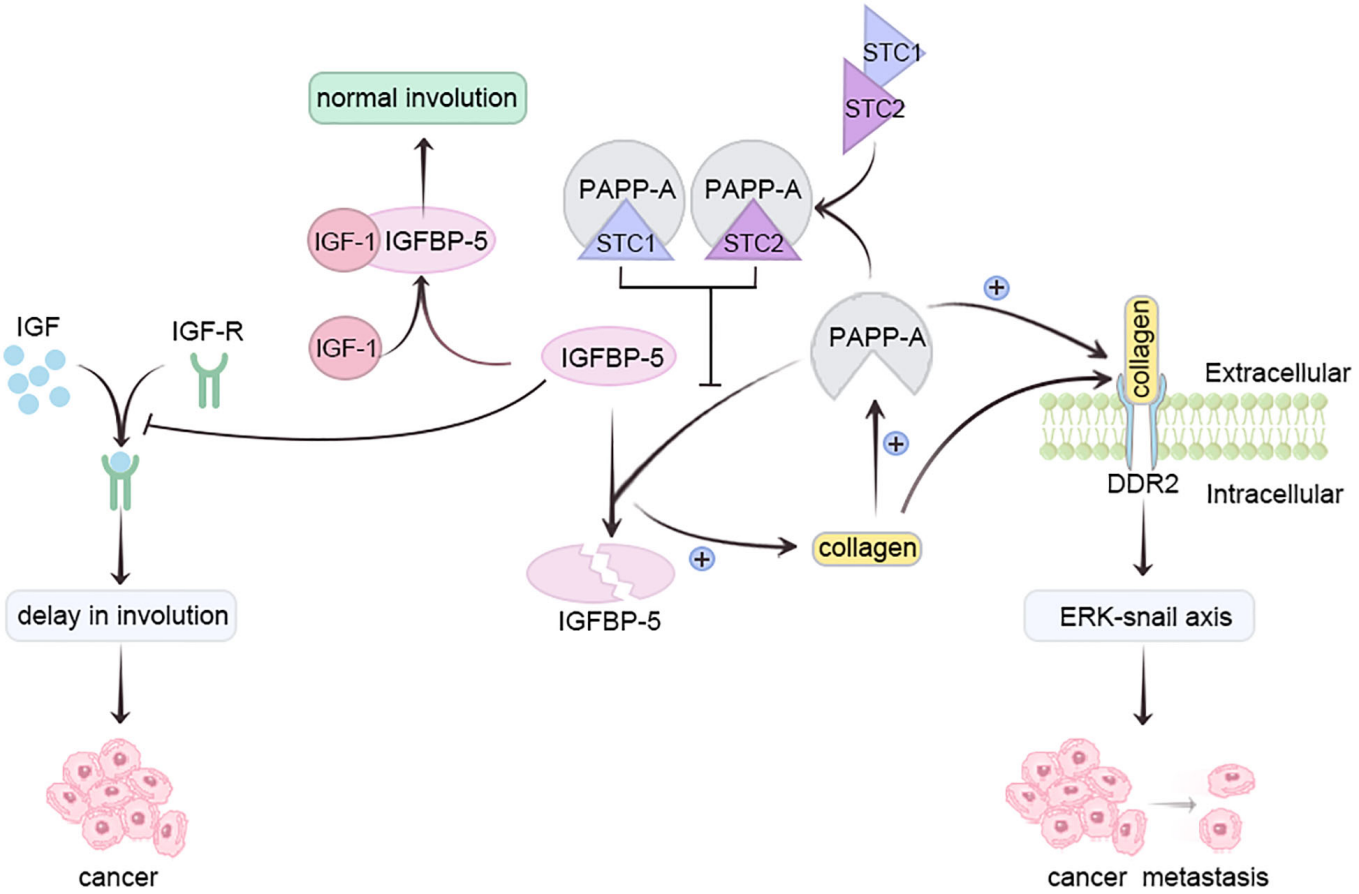
Prolonging breastfeeding duration can reduce the activity of PAPP-A through STC and inhibit the occurrence of breast cancer.

Moreover, PAPP-A activates collagen receptor DDR2, promoting tumor metastasis *via* ERK-Snail axis activation. Lack of breastfeeding triggers elevated collagen deposition during breast degeneration, amplifying PAPP-A activity (31, 35). Prolonged breastfeeding duration directly correlates with decreased PAPP-A activity during lactation (35). Glycoproteins Stanniocalcin-1 (STC1) and Stanniocalcin-2 (STC-2) inhibit PAPP-A, preventing PABC development; they are present in breast milk (35). STC, abundant during late pregnancy and lactation, diminishes post-lactation (36). Prolonged breastfeeding heightens STC1 and STC2 levels, inactivating PAPP-A, averting IGFBP-5 cleavage, allowing normal involution. Post-lactation, STC1 and STC2 levels decline, but PAPP-A activity doesn’t recover (35). *In vitro* studies confirm STC2’s PAPP-proteolytic A suppression through covalent bonding, curtailing PAPP-A-mediated IGF signaling (37). Conversely, STC1, lacking essential cysteine residue, binds PAPP-A with high affinity, sans covalent bonding (38). Absence of breastfeeding may hinder STC1 and STC2 from halting PAPP-A, fostering excess PAPP-A, IGFBP-5 cleavage, IGF signaling feedback, fostering PABC (**Figure 2**) (35).

## 4 Breastfeeding and the cellular microenvironment

Cellular surroundings profoundly influence growth and development. Breast milk markedly curbs cancer cells in the microenvironment. Throughout breastfeeding, calcium ion-rich breast milk inhibits cell apoptosis and necrosis (39, 40). Frequent exposure to elevated calcium ions can disrupt intercellular connections (41). Additionally, breast milk’s Secretory IgA (SIgA) and Lactalbumin Alpha (LALBA) compound suppress breast cancer cell development and induce apoptosis (42).

## 5 Breastfeeding and oncogenes

Oncogene BRCA1-IRIS, tied to breast cancer, arises from differential BRCA1 locus utilization. IRIS mRNA and protein exhibit significant upregulation in breast tumors (43), and normal breast tissue (44). IRIS overexpression spurs mammary progenitor cell growth, survival during gestational preparation for lactation (45). However, IRIS overexpression also leads to normal mammary epithelial cell differentiation into Triple Negative Breast Cancer (TNBC)-like cells (46). Beyond 12 months of breastfeeding, signaling pathways like VD/VDR/STAT3 elevate, diminishing IRIS expression, fostering mammary epithelial terminal differentiation (45). These cells also serve as tumor-specific peptide-presenting cells, cleared by immune cells upon breastfeeding cessation and involution. Inadequate breastfeeding might result in numerous IRIS-overexpressing progenitor cells during involution (47). Elshamy et al.’s “oncogene elimination hypothesis” elucidates this process (48). Known oncogenes possess normal functions at normal expression levels, sometimes overexpressed at developmental stages for specific roles (49). Breast cells’ initial gestational stage expression rise, sustaining survival, proliferation readiness for subsequent lactation. These cells must exit at breast degeneration’s onset, given inflammatory environment during degeneration. Differentiated cells might die, while progenitor cells thrive, turning more invasive (50). Prolonged breastfeeding induces terminal differentiation, clearing terminally-differentiated cells after involution onset (51). Inadequate breastfeeding might result in immune escape of IRIS-overexpressing progenitor cells during involution, fostering breast cancer development (48).

## 6 Breastfeeding and involution

Breast tissues undergoing involution after breastfeeding differ from those experiencing immediate involution after pregnancy without breastfeeding (52). During mammary gland involution, a few mammary epithelial cells revert to their pre-pregnancy state, while most undergo programmed death (53). However, the remodeling process varies based on breastfeeding duration. A prospective cohort study with mice revealed that the absence of breastfeeding leads to abrupt breast tissue remodeling, escalating inflammatory marker levels, and collagen deposition. *In vivo* mouse studies demonstrated that this sudden breast tissue remodeling results in substantial ductal hyperplasia, squamous metaplasia, and sustained elevation of luminal progenitor cells. Though unverified in human studies, these changes enhance cancer development potential (54). Following breastfeeding, breast tissues undergo involution, accompanied by a genetic signature expressing genes linked to apoptotic pathways like *p53*, *c-myc*, and *BCL-xl*. This expression enables efficient carcinogen metabolism and DNA damage repair (10). Inadequate breastfeeding sustains a terminal bud structure in breast tissue after involution, harboring numerous epithelial cells vulnerable to carcinogen stimulation, thereby facilitating breast cancer cell transformation (55).

## 7 Conclusion

**Figure 3:**
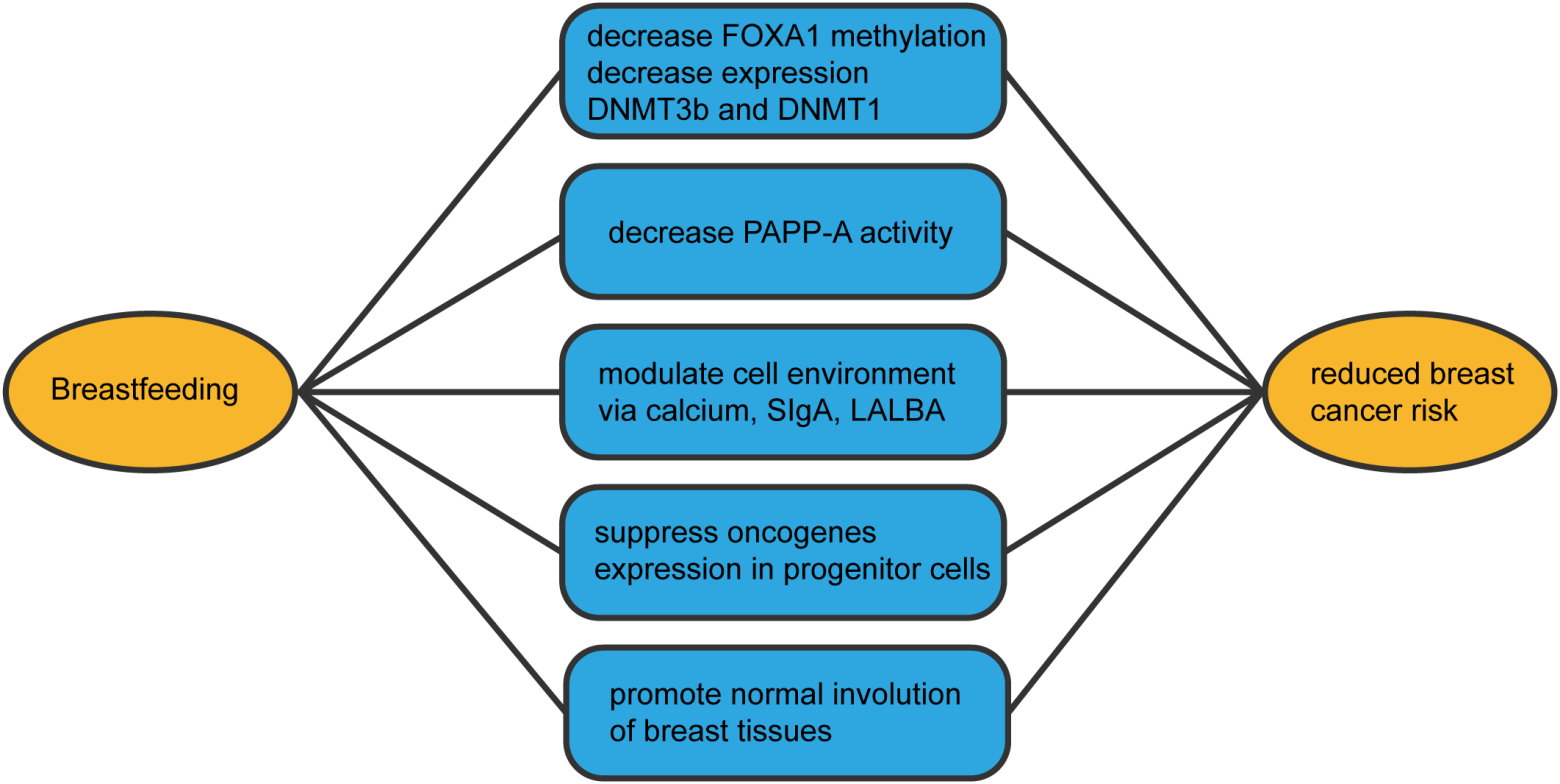
The mechanisms by which breastfeeding reduces the incidence of breast cancer.

Breastfeeding constitutes a positive, health-promoting behavior, with breastfeeding duration reducing breast cancer risk. Building on multiple breastfeeding-breast cancer associations, we comprehensively outlined mechanisms (**Figure 3**) through which breastfeeding averts breast cancer development. Numerous questions remain regarding breastfeeding’s role in reducing breast cancer risk. For instance, how much breastfeeding is required to mitigate risk? Is three months sufficient? Is the first or last pregnancy more pivotal, or is complete breastfeeding month duration key to risk reduction? Addressing these queries mandates extensive epidemiological studies providing detailed reproductive and breastfeeding histories, coupled with laboratory research illuminating these variables’ impact on breast cell populations. Future population-based inquiries must consider potential confounding effects of menarche age on breastfeeding associations, alongside possible interactions with other lifestyle factors such as oral contraceptive use, alcohol consumption, and body mass index. Enhanced understanding of breastfeeding’s impact on breast cancer mechanisms might uncover preventive pharmaceutical options for women unable or unwilling to breastfeed, curtailing cancer prevalence and its mortality, thereby setting our research’s future trajectory.

## Author contributions

YC: Conceptualization, Data curation, Resources, Writing – original draft. PJ: Data curation, Resources, Writing – review & editing. YG: Writing – review & editing.
